# Clinical Factors Associated With Patterns of Medication Errors Among Pediatric Hospitalized Patients in Northwest Ethiopia: A Multicenter Prospective Observational Study

**DOI:** 10.1155/bmri/8893135

**Published:** 2026-05-23

**Authors:** Tilaye Arega Moges, Fisseha Nigussie Dagnew, Getachew Yitayew Tarekegn, Samuel Agegnew Wondm, Woretaw Sisay Zewdu, Sisay Sitotaw Anberbr, Habtamu Nigussie, Samuel Berihun Dagnew

**Affiliations:** ^1^ Department of Clinical Pharmacy, Pharmacy Education and Clinical Services Directorate, Debre Tabor University, Debre Tabor, Ethiopia, dtu.edu.et; ^2^ Department of Pharmacy, College of Medicine and Health Sciences, Debre Markos University, Debre Markos, Ethiopia, dmu.edu.et; ^3^ Department of Pharmacology and Toxicology, Pharmacy Education and Clinical Services Directorate, Debre Tabor University, Debre Tabor, Ethiopia, dtu.edu.et; ^4^ Department of Pharmacy, College of Medicine and Health Sciences, Debre Berhan University, Debre Berhan, Ethiopia, dbu.edu.et

**Keywords:** Ethiopia, medication errors, medication safety, pediatric inpatients, polypharmacy

## Abstract

**Background:**

Medication safety is an important public health challenge, especially in pediatrics. Medication errors (MEs) are often underreported in pediatrics and can lead to adverse outcomes such as frequent readmissions, increased total healthcare costs, prolonged hospitalization, and related morbidity and mortality. Thus, this study is aimed at assessing the magnitude and determinants of MEs among pediatric hospitalized patients at comprehensive specialized hospitals in Northwest Ethiopia.

**Methods:**

A multicenter prospective observational study involving pediatric hospitalized patients was conducted over 4 months, utilizing systematic random sampling for participant selection. Three clinical pharmacists, after a day of training, collected data under the supervision of an MSc health professional, with support from pediatricians in each hospital for reviewing MEs and adjusting treatment plans. Pediatric patients were followed prospectively during their hospital stay from admission to discharge. Data collection occurred via the Kobo Toolbox platform and was analyzed with STATA Version 17.0. Both bivariate and multivariable logistic regression analyses identified factors related to MEs, with statistical significance set at a *p* value < 0.05.

**Results:**

Among 358 pediatric hospitalized patients, 53.63% experienced at least one ME, totaling 254 identified errors. The prescribing stage accounted for the highest percentage of errors (40.16%), followed by the administration stage (32.68%). The predominant types of MEs were dose errors (30.31%), frequency errors (14.96%), and omission errors (14.17%). Multivariable logistic regression analysis revealed that polypharmacy (≥ 5 medications) (AOR = 2.005, 95% CI: 1.269–3.168), male sex (AOR = 1.707, 95% CI: 1.097–2.656), and prolonged hospital stay (AOR = 1.673, 95% CI: 1.076–2.602) were significantly associated with the occurrence of MEs.

**Conclusion:**

This study found that MEs were prevalent in pediatric hospitalized patients. Polypharmacy, male patients, and the length of hospital stay were independent predictors of MEs. To reduce MEs, computer‐based prescribing practice and clinical pharmacy services should be routine practices in the study settings.

**Key Points**

• Medication errors (MEs) are underreported as a cause of preventable harm among hospitalized pediatric patients, particularly in low‐income countries.
• More than half of participants experienced at least one ME, underscoring a largely unrecognized patient‐safety burden.
• Prescription and administration stages accounted for the majority of errors, highlighting critical intervention points in pediatric medication management.
• Polypharmacy (≥ 5 medications), male gender, and hospital stay were associated with a higher risk of MEs.
• The study strengthened that computerized prescribing, involvement of clinical pharmacy, and medication review and reconciliation processes should be implemented to enhance pediatric care.

**Summary**

• Medication safety is especially important in children, who are more vulnerable to MEs. MEs can lead to longer hospital stays, higher costs, and poorer health outcomes.
• This study is aimed at assessing the magnitude of MEs among hospitalized children in Northwest Ethiopia and the factors that increase the risk.
• The study followed 358 pediatric patients admitted to pediatric wards over 4 months. More than half of the children (54%) experienced at least one ME, and a total of 254 errors were identified.
• Most errors happened during the prescription and administration stages. The most frequent errors were wrong doses, wrong timing, and omitted doses.
• Polypharmacy, male gender, and longer hospital stay were more likely to experience MEs.
• The study revealed that MEs are common among hospitalized children in this region. To reduce these errors, hospitals should strengthen computer‐based prescribing.

## 1. Introduction

MEs affect patient safety and are a serious public health concern [1]. MEs cause a large number of adverse drug events (ADEs) with negative patient treatment outcomes. They are a major public health concern and contribute to about 18.7%–56% of all ADEs among hospitalized patients [2]. In pediatrics, the presence of ADEs due to MEs was higher than in adults [3, 4], and this might be due to pediatrics’ physiological and developmental stage differences, including organ maturity, variation in weight, and age [5, 6]. These errors can occur at various stages, including prescription, administration, and monitoring, and can lead to adverse outcomes such as frequent readmissions, increased total healthcare costs, prolonged hospitalization, and related morbidity and mortality [7]. The World Health Organization (WHO) launched a global patient safety program, “Medication without Harm,” in March 2017, and the program seeks to reduce medication‐related harm by half in all countries within 5 years [8].

Various factors increase the occurrence of MEs, including older age, a poor healthcare system, a high number of prescribed drugs (polypharmacy), the presence of comorbidities, several prescribers for a single patient, and prolonged hospital stay, just to mention a few of them [6, 9–15]. MEs, in turn, contribute to various adverse health outcomes, such as drug–drug interactions, a higher number of hospital readmissions, prolonged hospital stays, elevated healthcare costs, and amplified patient mortality rate [16, 17].

Worldwide, about 5%–41.3% of all admissions to the hospital were due to MEs, and after discharge from hospitals, it accounts for 22% of readmissions [1]. In addition to directly affecting patients and the general public, MEs have potential consequences for healthcare professionals who commit errors, including patient mistrust; legal and civil actions, including criminal charges; and disciplinary actions by concerned authorities [18].

In Ethiopia, a nation with a rapidly developing healthcare system, pediatric care presents unique challenges. Medication management in pediatric patients can be complex due to variations in dosing requirements based on weight and age and the limited availability of pediatric‐specific formulations [11, 12, 14]. Moreover, healthcare facilities in Ethiopia, especially those in resource‐limited settings, may face constraints such as inadequate training for healthcare professionals, insufficient medication safety protocols, and limited resources [15, 19]. Despite the growing recognition of MEs as a critical issue in healthcare, there is limited research specifically addressing the prevalence and patterns of these errors among pediatric patients in Northwest Ethiopia. In particular, there is a lack of comprehensive data on how these errors manifest in pediatric wards at comprehensive specialized hospitals (CSHs) in Northwest Ethiopia and the factors that contribute to them.

To design effective strategies to prevent MEs and improve patient safety, an in‐depth understanding of the types of MEs occurring, their frequency, and the underlying causes is important [20]. Therefore, there is a pressing need to conduct a detailed prospective multicenter study on MEs among pediatric hospitalized patients at these facilities to inform local healthcare practices and policies. The findings will serve as valuable data for healthcare policymakers and administrators in Ethiopia, providing evidence to support the formulation of policies aimed at reducing MEs and improving healthcare delivery, and they may use this as input for developing targeted strategies to enhance patient safety. It will also highlight areas needing further research, fostering a culture of continuous improvement in pediatric healthcare.

## 2. Methods and Materials

### 2.1. Study Design, Study Period, and Setting

The present study was a multicenter prospective observational study that was conducted in pediatric hospitalized patients at public CSHs in Northwest Ethiopia over 4 months (February 1 to May 30, 2024). CSHs in Northwest Ethiopia include the University of Gondar CSH, Debre Tabor CSH, Felege Hiwot CSH, Tibebe Ghion CSH, and Debre Markos CSH. The present study recruited three hospitals by lottery method: the University of Gondar CSH which is located 750 km northwest of Addis Ababa and serves more than 7 million people in the Amhara Region; Debre Tabor CSH, which is located at a distance of 667 km from the capital city, Addis Ababa, and 104 km from Bahirdar City and provides service for more than 3 million people in the area; and Felege Hiwot CSH, which is located in Bahir Dar City, the capital of the region, which is 565 km from Addis Ababa, and serves about 5 million populations.

### 2.2. Eligibility Criteria

The population source was all pediatric patients admitted to each hospital’s pediatric wards in Northwest Ethiopia. The study population comprised all pediatric patients who were admitted to pediatric wards of each hospital during the study period and who fulfilled the inclusion criteria. Pediatric patients admitted to the pediatric wards of each hospital and stayed for more than 24 h, diagnosed with at least one medical disease condition, and received at least one medication and pediatric inpatients whose parent/guardian signed the written informed consent were included in the study. Pediatric inpatients who were readmitted, patients who stayed less than 24 h, and those who did not sign a written informed consent were excluded.

### 2.3. The Study Variables

The study focused on MEs, defined as any error occurring during the medication‐use process (prescription, transcription, dispensing, administration, and patient use). Identifying these errors involved reviewing prescriptions, observing medication handling, and assessing patient medication use. The composite outcome variable included different types of MEs as defined in previous studies [13, 19, 21–23]:

• Dispensing error: This is defined as preparation and manipulation of medications incorrectly, like incorrect reconstitution method, suspensions administered without the counseling aid of shaking thoroughly, and crushing of coated tablets.
• Dose error: This is defined as a dose that was prescribed or administered > 10% above or below the correct dose based on the patient’s weight.
• Incorrect patient action (compliance errors): This is defined as MEs, such as not following protocol or rules established for dispensing and prescribing medications. Patient education is the only way to prevent this type of error.
• Drug selection error: This is defined as selecting the wrong drug for the correct diagnosis, prescribing drugs that are contraindicated in a patient, and drug duplication.
• Duration error: This is defined as medication administered for a longer period than was prescribed or prescribed medication that was not discontinued when indicated.
• Frequency error: This is defined as administering medications at incorrect intervals (e.g., “Bid,” which is every 12 h instead of every 8 h [tid]; “Qid,” which is every 6 h instead of every 4 h; and “Qd,” which is every day instead of four times a day [Qid]).
• Administration error: This is defined as medication administration not yet prescribed, administering it to the wrong patient, or misreading the medication written on the prescription.
• Omission error: This is defined as missing medication that was intended for the patient and the prescribed medication that was not given or taken by the patient.
• Prescribing error: This is defined as AN error that was not adhering to elements of good prescribing practice (GPP) and as specified in good pharmacy practice by evaluating prescription components like drug name, correct dose, frequency, route of administration, duration of therapy, and units of dose measurement.
• Wrong route: This is defined as the wrong route on the prescription and administering medication to a patient through an incorrect route of administration than was prescribed.
• Medication transcription error: This type of error occurs when there is a discrepancy between a physician’s original order and what is recorded on a patient’s profile, medication administration record (MAR), or pharmacy system. Common causes include illegible handwriting, similar drug names, and fatigue, leading to errors such as incorrect dosages, wrong administration routes, or omitted medications.
• ME: This is defined as an occurrence of at least one of the abovementioned MEs at any stage of the error.

The explanatory variables include gender, weight, diagnosis, family members’ involvement in child care, length of hospital stay, the number of drugs taken during the hospital stay, medication preparation room evaluation (e.g., availability of calculator machine), protocol for medication administration, standard weight measurement, interruption during administration of medications, documentation system, and all medicine‐related variables.

### 2.4. Sample Size Determination and Sampling Technique

To estimate the sample size required for the study, the following single population proportion formula was used:

n=z2p1−pd2,

where *n* is the required sample size, *z* is the confidence level of 95%, *p* is the estimated magnitude of ME from the previous study, and *d* is the estimated margin of sampling error with the assumption of 95% confidence interval (CI), marginal error (*d*) of 5%, *Z* *α*/2 = 1.96, and *p* = 69.5*%* of pediatric inpatients who experienced MEs in public hospitals of Gamo Zone, Southern Ethiopia [14]:

n=z21−ppd2,

n=1.96210.695−0.6950.052,n=325.72326=.

**Figure 1:**
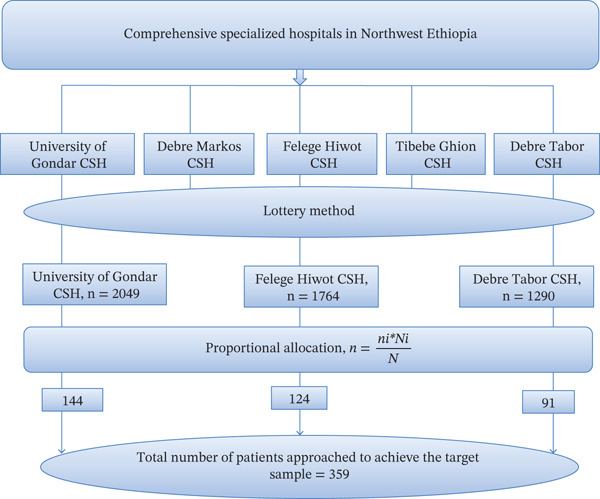
Diagrammatic representation of the sampling procedure among public comprehensive specialized hospitals in Northwest Ethiopia.

By considering a 10% nonresponse rate, the final sample size becomes 359. Then, the proportional allocation of samples to the total population of each hospital’s pediatric ward was applied using the formula as follows (Figure 1):

n=ni∗NiN,

where *n* is the total sample size to be selected, *N* is the total population, Ni is the total population of each hospital’s pediatric ward annually, and ni is the sample size from each hospital’s pediatric ward.

University of Gondar (*n*1 = (359∗2049)/5103 = 144), Debre Tabor (*n*2 = (359∗1290)/5103 = 91), and Felege Hiwot (*n*3 = (359∗1764)/5103 = 124). Total sample size = *n*1 + *n*2 + *n*3 = 359. A total of 358 pediatric inpatients were involved, and among 359 patients approached to achieve the target sample, in the present study, with a response rate of 99.72%, one patient was withdrawn because of unwillingness to give informed consent in the study. A systematic random sampling technique was used to involve patients in the present study. The sampling interval (*k*) was taken by dividing the total annual pediatric patient admission by the total sample size, 5103/359 = ~14. The first patient was selected by lottery method; three (3) were taken at random and used as a sampling interval. Then, based on their admission order, patients were selected at intervals of three until the target achievable sample size was achieved.

### 2.5. Data Collection and Management Methods

In the present study, a structured questionnaire was developed after reviewing previously conducted studies [6, 11, 13–15, 24–26]. Three clinical pharmacists, after having 1 day of training on the study’s objectives, data collection techniques, and procedures, collected the data, and one MSc health professional supervised the study. In each hospital, a pediatrician was assigned as the responsible person for the present study to adjust the treatment plan of pediatric inpatients if an error was encountered. Instructions on how to obtain the most relevant data about MEs during the interview with the patient’s parents/caregivers were discussed with the nursing staff of each hospital. Data collectors, the clinical pharmacists, visited the pediatric wards of each hospital to collect the data using a structured and pretested questionnaire and record all relevant information required to characterize the detected MEs. The clinical pharmacists and the pediatrician responsible at each hospital evaluated the MEs that were detected. The patient was followed from admission to discharge.

Detected MEs were classified using the National Coordinating Council for Medication Error and Prevention (NCCMERP) Index [27]. One week before the actual data collection, the tool was pretested on 5% of patients (18 inpatients) at Debre Markos CSH for its consistency and clarity, and the tool was used without modifications since there were no consistency or clarity problems. The procedures for assessing the validity and reliability of the data collection tool were performed. The type of validity assessment conducted was face/content validity, and the method used to evaluate internal consistency included Cronbach’s alpha (0.72 in the present study). Data collection procedures included interviews of parents/caregivers; observation of the nurses during dilution, reconstitution, and medication administration; and extraction of the patient’s medical records. The data collection process was regularly checked, and data collectors were assisted by the supervisor to ensure data quality and consistency of the response. Any error identified has been corrected immediately after the interview is completed by communicating with the treating physician and pediatrician at the pediatric wards of each hospital.

### 2.6. Statistical Analysis and Data Processing

The data was collected by the Kobo toolbox and exported to Stata Version 17.0 for analysis, after checking the data quality, including missing values. Descriptive analyses such as frequencies, mean, interquartile range, and standard deviation (SD) were used to describe the study participants’ descriptive results. To explore the association between patients with and without MEs, a chi‐square test (*χ* ^2^ test) was used for categorical variables. Binary logistic regression analysis was used to examine the association between explanatory variables and the dependent variable, ME. Explanatory variables with a *p* value of less than 0.25 in the bivariate logistic regression analysis were included in the multivariable logistic regression analysis. Multicollinearity was checked using the variance inflation factor (VIF) (all variables’ values less than 5) and tolerance test (variables with values above 0.2). Model goodness of fit was assessed by the Hosmer–Lemeshow test (chi − square = 3.787, Sig. = 0.876).

Finally, a crude odds ratio (COR) and adjusted odds ratio (AOR) with a 95% CI were computed, and a CI that did not cross or contain the value 1 and variables with a *p* value of < 0.05 were considered statistically significant determinant factors of MEs.

## 3. Results

### 3.1. Baseline Characteristics of the Study Participants

In the present study, a total of 358 pediatric inpatients were recruited, with a response rate of 99.72% (358/359), and 186 (52.0%) were male. For the majority of patients, diagnosis was done based on both empirical and laboratory (281, 78.5%). The majority of pediatric hospitalized patients, 297 (82.96%), in this study weighed 5 and 19 kg, and the mean weight (±SD) of patients was 10.24 kg (±7.8). Two hundred and seven (57.8%) pediatric patients stayed more than 5 days in the hospital with a mean duration (±SD) of 8.46 days (±4.21). Approximately 296 (82.7%) of patients were newly admitted to the pediatric wards of CSHs in Northwest Ethiopia, and 147 (41.1%) patients were taking a high number of medications (≥ 5 medicines). About 305 (85.2%) of pediatric patients had no previous history of medical illness. The most common reason for pediatric inpatients’ admission to the hospital was pneumonia, 80 (22.4%), followed by severe acute malnutrition, 66 (18.4%) (SAM); meningitis, 63 (17.6%); and urinary tract infection, 56 (15.6%) (Table 1).

**Table 1:** Baseline characteristics of pediatric hospitalized patients at comprehensive specialized hospitals in Northwest Ethiopia (*n* = 358).

Variables	Category	Frequency	Percent
Gender	Male	165	46.1
	Female	193	53.9

Residence	Urban	206	57.5
	Rural	152	42.5

Weight of child (in kg)	< 5	8	2.2
	5–19	297	83.0
	≥ 20	53	14.8

Availability of medicine	Available	278	77.7
	Not available	80	22.3

Level of consciousness	Conscious	305	85.2
	Unconscious	53	14.8

Previous history of medical illness	Yes	58	16.2
	No	300	83.8
Diagnosis criteria	Empirical	45	12.6
	Laboratory investigation	32	8.9
	Both empirical and laboratory	281	78.5

Number of diseases per patient	< 3	210	58.7
	≥ 3	148	41.3

Number of medications per patient	< 5	211	58.9
	≥ 5	147	41.1

Hospital stays in days	≤ 5	151	42.2
	> 5	207	57.8

Type of admission	New	296	82.7
	Transferred	62	17.3

Reasons for admission	Malaria	8	2.2
	Sepsis	12	3.4
	Pneumonia	80	22.4
	Anemia	13	3.6
	Meningitis	63	17.6
	Severe acute malnutrition	66	18.4
	Low birth weight	9	2.5
	Urinary tract infection	56	15.6
	Abscess	10	2.8
	Diarrhea	21	5.9
	Dehydration	14	3.9
	Others^a^	6	1.7

^a^Acute gastroenteritis, scabies, burn, prematurity, and traditional uvulectomy.

### 3.2. Standard Prescription‐Related Characteristics Among Pediatric Inpatients

In the present study, prescriptions were evaluated based on GPPs. It was noted that almost all of the prescriptions missed at least one essential component based on the recommended national guidelines, showing nonadherence of prescribers to good prescription practices, and this may lead to irrational medication use. Medical diagnosis, 67 (18.7%); patients’ weight, 63 (17.6%); duration of therapy, 53 (14.8%); patient address, 50 (13.4%); and patient gender, 45 (12.6%), were the most commonly missed components on the prescriptions (Table 2).

**Table 2:** Standard prescription‐related characteristics among pediatric hospitalized patients at comprehensive specialized hospitals in Northwest Ethiopia (*n* = 358).

Prescription‐related characteristics	Frequency	Percent
Medical registration number	222	62.0
Patient’s full name	349	97.5
Patient age (in years)	220	61.5
Patient address	50	13.4
Patient gender	45	12.6
Weight of the patient (in kg)	63	17.6
Diagnosis	67	18.7
Date of prescription	338	94.4
Drug name	348	97.2
Drug dose	350	97.8
Frequency of administration	329	91.9
Route of administration	327	91.3
Duration of therapy	53	14.8
Name and signature of the prescriber	161	44.7
Legible handwriting of the prescriber	71	19.8
Unstandardized abbreviations	230	64.2

### 3.3. Magnitude and Types of MEs Among Pediatric Inpatients

Among 358 pediatric hospitalized patients, about 53.63% of patients experienced MEs (192/358). During the study period, a total of 254 MEs were detected in 192 patients. The most commonly encountered stages with MEs were the prescription, 102 (40.16%), and medication administration, 83 (32.68%), stages. The common type of ME in this study was dosing error, 77 (30.31%), followed by frequency error, 38 (14.96%), and omission error, 36 (14.17%) (Table 3).

**Table 3:** Magnitude, types, and stages of medication errors among pediatric hospitalized patients at comprehensive specialized hospitals in Northwest Ethiopia (*n* = 358).

Variable	Category	Frequency	Percent
Type of medication error (*n* = 254)	Dose error	77	30.31
	Wrong route	25	9.84
	Frequency error	38	14.96
	Omission error	36	14.17
	Duration of therapy error	27	10.63
	Error in drug selection	20	7.87
	Reconstitution error	22	8.66
	Incorrect patient action	9	3.54

Stage of medication error (*n* = 254)	Prescribing	102	40.16
	Drug administration	83	32.68
	Dispensing	47	18.50
	Monitoring	22	8.66

The magnitude of medication error per patient (*n* = 358)	Yes	192	53.63
	No	166	46.37

### 3.4. Therapeutic Classes of Medications Associated With MEs

**Figure 2:**
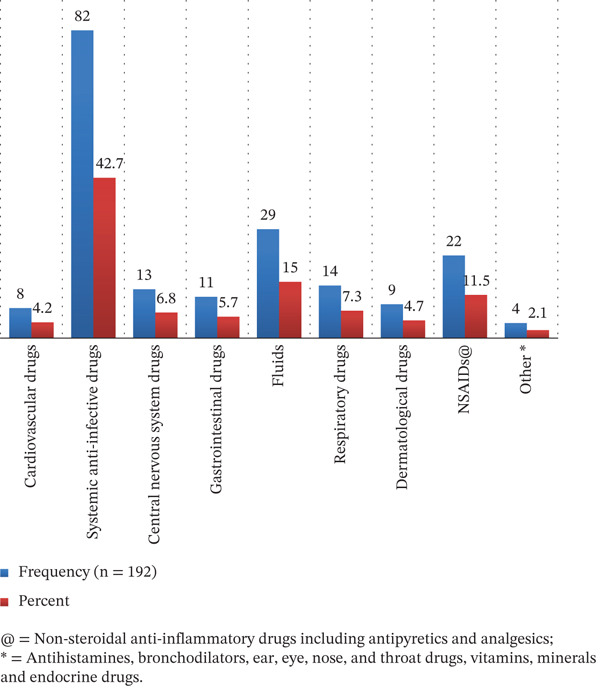
Therapeutic classes of drugs involved in medication errors among pediatric inpatients in Northwest Ethiopia, 2024.

Among 192 patients who ended up with MEs, different drug classes were involved with MEs. Systemic anti‐infective drugs, 82 (42.7%), followed by fluids, 29 (15.0%), and NSAIDs, 22 (11.5%), were the classes of medications most commonly involved in MEs (Figure 2).

### 3.5. Factors Contributing to the Occurrence of MEs

After controlling for confounding variables, the three variables that were significantly correlated with MEs were the number of medicines per patient, male gender, and longer hospital stays. MEs were found to be independently correlated with male gender; pediatric male patients were 1.71 times more likely than female patients to make MEs (AOR = 1.707, 95% CI: 1.097, 2.656). Compared to their counterparts (< 5 medicines), pediatric patients with polypharmacy (≥ 5 medicines) had a twofold (AOR = 2.005, 95% CI: 1.269, 3.168) higher odds of experiencing MEs. Compared to those with fewer hospital stays (≤ 5 days), those with longer hospital stays (> 5 days) have 1.673 times (AOR = 1.673, 95% CI: 1.076, 2.602) higher chance of MEs (Table 4).

**Table 4:** Bivariate and multivariable logistic regression analysis of predictors of medication errors among pediatric inpatients in Northwest Ethiopia, 2024.

Variables	Category	Medication error		COR (95% CI)	AOR (95% CI)	*p*value
		No	Yes			
Gender	Female	101	92	1	1	0.018 ^∗^
	Male	65	100	1.689 (1.109, 2.573)	1.707 (1.097, 2.656)	
Residence	Urban	89	117	1	1	0.268
	Rural	77	75	0.741 (0.486, 1.129)	0.780 (0.502, 1.211)	
Type of admission	New	142	154	1	1	0.224
	Transferred	24	38	1.460 (0.834, 2.555)	1.442 (0.800, 2.599)	
Number of medicines per patient	< 5 medicines	110	101	1	1	0.003 ^∗^
	≥ 5 medicines	56	91	1.770 (1.153, 2.717)	2.005 (1.269, 3.168)	
Availability of medicine	Available	123	155	1	1	0.129
	Not available	43	37	0.683 (0.414, 1.125)	0.668 (0.398, 1.124)	
Length of hospital stays	≤ 5 days	80	71	1	1	0.022 ^∗^
	> 5 days	86	121	1.585 (1.039, 2.419)	1.673 (1.076, 2.602)	
Level of consciousness	Conscious	136	169	1	1	0.205
	Unconscious	30	23	0.617 (0.343, 1.111)	0.674 (0.366, 1.240)	
Number of diseases per patient	< 3 diseases	104	106	1	1	0.328
	≥ 3 diseases	62	86	1.361 (0.891, 2.080)	1.246 (0.801, 1.938)	

*Note:* Reference [1].

Abbreviations: AOR, adjusted odds ratio; COR, crude odds ratio.

^∗^Statistically significant at a *p* value < 0.05.

## 4. Discussion

Pediatric inpatients are more vulnerable to MEs, predominantly because of the need for multifaceted dosage calculations, which are individually based on the patient’s age, weight, body surface area, and medical conditions [28]. MEs’ incidence and clinical outcomes like severe disability and death are common and more harmful in pediatrics than in adults [9, 10, 29]. In resource‐limited countries, like Ethiopia, there is limited data regarding MEs among pediatric hospitalized patients, particularly in the northwestern part of Ethiopia. Thus, this multicenter prospective observational study is aimed at assessing the magnitude and determinants of MEs among pediatric hospitalized patients at CSHs in Northwest Ethiopia.

In the present study, 53.6% (95% CI: 48.3, 58.9) of pediatric hospitalized patients were prone to MEs. This study’s finding was higher than the study in Southwest Ethiopia (41.8%) [11]. However, this study finding was lower than the study conducted in West Ethiopia (75.1%) [19], Northern Ethiopia (62.7%) [13], Southern Ethiopia (69.5%) [14], and South Africa (78%) [30]. These discrepancies may be due to work overload, the training and experience of healthcare professionals, the availability of training focused on medication safety, the infrastructure of the facilities, the involvement of clinical pharmacists in the healthcare team, medication administration guidelines, and electronic prescription practice and dose calculation machines. Studies have demonstrated that hospitals with electronic prescribing systems and clinical decision support tools have significantly lower ME rates compared to facilities relying on handwritten prescriptions [31, 32]. In addition, the involvement of clinical pharmacists in multidisciplinary healthcare teams has been shown to reduce MEs through medication review and dose verification services [33–35].

Among 254 types of MEs detected in the present study, the most common stage of ME was prescription error (40.16%), and regarding the type of ME, the most common error was dosing error (30.31%), followed by frequency of administration error (14.96%) and omission error (14.17%). Incorrect dosing, routes, frequency of administration, and other errors often result in morbidity and mortality [25, 36]. In a present study, about 3.54% of MEs were due to incorrect patient action. Patients incorrectly taking medication is considered a ME, and it is an adherence error, such as not following protocol or rules established for dispensing and prescribing medications [26]. Patient education is the only way to prevent this type of error [21, 26]. Monitoring errors account for about 8.66% of MEs and occur at the stage of monitoring, such as failing to take into account the patient’s liver and renal function and failing to document allergy or any drug interactions [26].

Regarding medication prescription‐related characteristics, the present study showed that almost all of the prescriptions missed at least one standard prescription form component, which is suggested by national GPPs/guidelines. Medical diagnosis, 67 (18.7%); patients’ weight, 63 (17.6%); duration of therapy, 53 (14.8%); patient address, 50 (13.4%); and patient gender, 45 (12.6%), were the predominant components/information missed in the prescriptions. This study’s findings were in line with the studies conducted so far [14, 37]. This study also revealed that about 19.83% of medication‐related prescriptions were illegible handwriting. The illegible handwriting of prescribers has plagued both nurses and pharmacists and the healthcare system for decades [38]. The Institute of Safe Medication Practices recommends stopping handwritten orders and prescriptions [24]. The possible justification for the illegible writing in this study is the absence of electronic prescriptions, a large number of patients, the absence of continuous training, and the lack of a strong system for monitoring and evaluation of encountered MEs.

In the present study, antibiotics were the most common class of drugs responsible for MEs, which was in line with a study conducted in western Ethiopia [15], in Southwest Ethiopia [11], Palestine [39], and Spanish public hospitals [6]. This might be because infectious diseases are the top medical conditions diagnosed in pediatric wards of resource‐limited countries that require anti‐infective medication therapy [40]. This reported that antimicrobial stewardship (AMS) interventions, which are effective tools for improving antibiotic prescribing for hospital inpatients [41], should be fully implemented in hospitals, and clinical pharmacists’ involvement should be encouraged in this initiative since the implementation of AMS enhances the regular surveillance of antibiotic usage at the patient level and antibiotic prescription practice at the institutional level.

The present study identified several significant factors associated with MEs among pediatric patients. After controlling for potential confounding variables, three main factors emerged as significantly correlated with MEs: the number of medications per patient, male gender, and the length of hospital stay. This study reported that a high number of medications (polypharmacy) significantly increases the occurrence of MEs. Pediatric patients receiving five or more medications had a twofold higher odds of experiencing MEs (AOR = 2.005, 95% CI: 1.269, 3.168) compared to those on fewer medications, and this is in line with the study done in western Ethiopia [15, 19]. The complexity of managing multiple medications can lead to increased opportunities for errors, such as incorrect dosages or drug interactions [21]. This finding underscores the importance of careful medication management and the need for strong interventions to reduce the risk of MEs in patients with complex medication regimens.

Length of hospital stay was another important factor that impacted the ME. Patients with hospital stays longer than 5 days had a 1.673 times higher chance of encountering MEs (AOR = 1.673, 95% CI: 1.076, 2.602) compared to those with shorter stays. This study was consistent with the previous studies [14, 19]. Longer hospital stays often involve more frequent medication administration and increased patient complexity, which may contribute to a higher risk of MEs, and interventions should be undertaken to reduce hospital length of stay [42]. This shows the need for review of medication and attentive monitoring, especially in patients with prolonged hospitalizations.

### 4.1. Strengths and Limitations of the Study

The study utilized a prospective observational design to effectively identify MEs throughout various stages of the medication‐use process, resulting in enhanced error identification accuracy and minimized recall bias. It employed established operational definitions to improve findings’ validity and comparability, and the data collection tool was validated before the study, ensuring data reliability. However, limitations include the study’s narrow healthcare setting scope, which may limit the generalizability of results, and potential underreporting of errors related to patient compliance outside the healthcare environment.

### 4.2. Conclusion and Recommendations

The magnitude of MEs was high in pediatric hospitalized patients in the study areas. In this study, MEs were most commonly identified at the prescription and administration stage. Dosing errors and frequency of administration errors were the most commonly encountered types of MEs detected in the present study. Antibiotics were the main class of drugs involved in MEs. Polypharmacy, male gender, and length of hospital stay were the independently associated factors of MEs in the study areas. Therefore, to reduce MEs, electronic prescriptions should include updated clinical practice guidelines in drug selection and the inclusion of drug details like drug dose, route of administration, frequency of administration, and duration of therapy. On‐the‐job training of healthcare providers should be implemented in the hospitals, and clinical pharmacists’ involvement in the direct patient care responsibility should be encouraged to optimize medication regimens, reduce medication‐related morbidity and mortality, and, hence, improve patient treatment outcomes.

**Nomenclature**
ADEs: adverse drug events
AOR: adjusted odds ratio
COR: crude odds ratio
CSH: comprehensive specialized hospital
MEs: medication errors
NCCMERP: National Coordinating Council for Medication Error Reporting and Prevention
WHO: World Health Organization

## Author Contributions

T.A.M.: conceptualization, data curation, formal analysis, methodology, software, supervision, investigation, project administration, writing original draft, and writing—review and editing; S.B.D. and H.N.: data curation, formal analysis, methodology, software, supervision, investigation, project administration, and writing—review and editing; F.N.D., S.A.W., G.Y.T., S.S.A., and W.S.Z.: data curation, formal analysis, investigation, methodology, supervision, and writing—review and editing.

## Funding

This research was conducted without specific grants from any funding agencies and was part of the authors’ academic responsibilities. The employer did not influence any aspect of the study, including design, data collection, analysis, manuscript preparation, or publication decisions.

## Ethics Statement

Ethical clearance was obtained from the ethical review committee of Debre Tabor University, College of Health Sciences (reference number: DTU/CBE/297/2024). Written informed consent was obtained from the parents or legal guardians after explaining the purpose of the study. The data were kept confidential. All methods were conducted based on the Helsinki Declaration.

## Consent

The authors have nothing to report.

## Conflicts of Interest

The authors declare no conflicts of interest.

## Data Availability

The data that support the findings of this study are available from the corresponding author upon reasonable request.
